# Pioneering gut health improvements in piglets with phytogenic feed additives

**DOI:** 10.1007/s00253-023-12925-2

**Published:** 2024-01-17

**Authors:** Sung Joon Yu, Andrew Morris, Advait Kayal, Ivan Milošević, Thi Thu Hao Van, Yadav Sharma Bajagai, Dragana Stanley

**Affiliations:** 1 Central Queensland Innovation and Research Precinct (CQIRP), Institute for Future Farming Systems, Central Queensland University, Rockhampton, QLD 4701 Australia; 2 Riverbend Pork Group, 487-489 Ruthven Street, Toowoomba, 4350 Australia; 3 Faculty of Veterinary Medicine, University of Belgrade, Bulevar Oslobodjenja 18, 11999 Belgrade, Serbia; 4 School of Science, RMIT University, Bundoora, VIC 3083 Australia

**Keywords:** Phytogenic feed additives, Gut microbiota, Intestinal morphology, Metagenomics, Metabolic pathways, Antibiotic alternatives

## Abstract

**Abstract:**

This research investigates the effects of phytogenic feed additives (PFAs) on the growth performance, gut microbial community, and microbial metabolic functions in weaned piglets via a combined 16S rRNA gene amplicon and shotgun metagenomics approach. A controlled trial was conducted using 200 pigs to highlight the significant influence of PFAs on gut microbiota dynamics. Notably, the treatment group revealed an increased gut microbiota diversity, as measured with the Shannon and Simpson indices. The increase in diversity is accompanied by an increase in beneficial bacterial taxa, such as *Roseburia*, *Faecalibacterium*, and *Prevotella*, and a decline in potential pathogens like *Clostridium *sensu stricto* 1* and *Campylobacter*. Shotgun sequencing at the species level confirmed these findings. This modification in microbial profile was coupled with an altered profile of microbial metabolic pathways, suggesting a reconfiguration of microbial function under PFA influence. Significant shifts in overall microbial community structure by week 8 demonstrate PFA treatment’s temporal impact. Histomorphological examination unveiled improved gut structure in PFA-treated piglets. The results of this study indicate that the use of PFAs as dietary supplements can be an effective strategy, augmenting gut microbiota diversity, reshaping microbial function, enhancing gut structure, and optimising intestinal health of weaned piglets providing valuable implications for swine production.

**Key points:**

• *PFAs significantly diversify the gut microbiota in weaned piglets, aiding balance*.

• *Changes in gut structure due to PFAs indicate improved resistance to weaning stress*.

• *PFAs show potential to ease weaning stress, offering a substitute for antibiotics in piglet diets*.

**Supplementary Information:**

The online version contains supplementary material available at 10.1007/s00253-023-12925-2.

## Introduction

The weaning period is recognised as a critical phase in pig management, characterised by a multitude of stressors, including environmental, social, and dietary changes that can negatively impact piglets’ health and growth and can cause reduced feed intake, weight loss, and an increased risk of diarrhoea and mortality (Heo et al. 2013; Lallès et al. 2004). These changes also significantly affect the piglet’s gastrointestinal tract (GIT), particularly the intestines, affecting nutrient digestion and absorption (Pluske et al. 1997). Alterations in the GIT structure and function are commonly observed after weaning. These include changes in villus height, crypt depth, and intestinal permeability, impairing nutrient utilisation and promoting inflammation (Spreeuwenberg et al. 2001). The gut microbiota, comprising diverse microorganisms, also undergo disturbances during weaning, potentially contributing to gastrointestinal issues and pathogenic infections (Gresse et al. 2017).

In pigs, the hindgut is the primary site for microbial fermentation, exhibiting higher microbial diversity than the less diverse small intestine (Kelly et al. 2017). The small intestine, responsible for nutrient absorption, is susceptible to dietary influences and is frequently exposed to various antigens and microbial components from the feed. The small intestinal mucosa encounters various exogenous antigens and microbial components from feed ingredients, and alteration in the mucosa-associated microbiota can significantly impact host growth and development (Niu et al. 2015).

During the weaning transition, there is a noticeable shift in the dominant microbial genus from *Bacteroides* to *Prevotella* and a reduction in *Lactobacilli* (Alain et al. 2014; Konstantinov et al. 2006). To mitigate weaning-associated intestinal dysfunction and impaired growth, effective dietary strategies such as using feed additives are being explored.

Phytogenic feed additives (PFAs) have gained tremendous attention because of their diverse biological functions, such as improving feed palatability, stimulating digestive enzyme secretion, modulating the gut microbiota, and possessing antioxidant, anti-inflammatory, and antimicrobial properties (Blavi et al. 2016; Windisch et al. 2008). Previous studies have demonstrated the positive effects of PFA in sow diets during late gestation and lactation, resulting in higher feed intake and growth rate in the post-weaning piglets (Blavi et al. 2016). The study by Li et al. (2012) evaluating the effects of essential oils containing thymol and cinnamaldehyde, supplemented in nursery pig feeds, demonstrated enhanced growth performance during the post-weaning period, comparable to antibiotic supplementation. The essential oil supplementation also improved dry matter and crude protein digestibility.

Apart from these factors addressed previously, sex of the animal has been recognised as a significant element influencing the changes in gut microbiota composition (Valeri and Endres 2021). Recent research investigating the sex-specific association between the gut microbiome and diet-induced metabolic disorders in mice revealed that sex exerted a greater impact on the composition of gut microbiota than environmental factors (Peng et al. 2020). Moreover, sex-specific differences in the immune system, independent of microbiota, contribute to the establishment of a sex-specific gut microbiota composition, further emphasising the role of sex in shaping the gut microbiota (Fransen et al. 2017).

There is a notable research gap regarding the use of PFAs to address post-weaning challenges in pig production, including diarrhoea, mortality, weight loss, and gut microbiota disturbances. Understanding the impact of dietary intervention, particularly PFAs, on post-weaning gut health and growth performance is crucial for developing effective strategies. Here, we present the effects of a PFA on the intestinal microbiota profile, microbial metabolic function, and performance of weaned piglets.

## Materials and methods

### Ethical statement

The study received approval from the institutional Animal Ethics Committee of Central Queensland University under reference number 0000023290. All animal procedures adhered to the guidelines outlined in the Australian Code for the Care and Use of Animals for Scientific Purposes. The study’s findings were reported in accordance with the Animal Research: Reporting of In Vivo Experiments (ARRIVE) guidelines and regulations.

### Animal trial

The study was performed on a selected pig farm to assess the effects of phytogenic supplements on the productivity of nursery pigs aged 3 to 8 weeks (Wk). Upon arrival at the farm, 200 pigs (Landrace × Large White), consisting of equal numbers of males and females, were randomly assigned into two treatment groups (control (Ctr) and treatment (Phy)) immediately after weaning at week 3. The pigs received unlimited access to water and solid feed. The control group received the basal diets consisting of barley, sorghum, wheat, soya meal, and meat and bone meal (MBM), formulated by company nutritionists to meet production and animal welfare requirements. The standard trace minerals, vitamins, organic acids, and pre- and probiotics were supplemented with the feed. The treatment groups received the same basal diet supplemented with a commercial PFA containing a blend of essential oils (rosemary, curcuma, and garlic), mucilages, and flavonoids, administered at a dosage of 1 kg per tonne of feed. The PFA supplementation commenced 1 week post-acclimatisation following the arrival of the animals and continued for 5 weeks.

Pigs were then marked for ongoing monitoring throughout the nursery stage. Upon arrival at the farm and again at week 8, individual body weights were recorded. Performance parameters such as general health, mortality, and body weight were monitored throughout the trial. For 16S rRNA gene amplicon analysis, rectal swab samples were collected at weeks 3, 5, and 8 using sterile cotton swabs, and the collected faecal samples were immediately stored on dry ice and preserved at − 80 °C until DNA extraction. It is important to note that, due to operational restrictions and the farm’s established routines, the acquisition of weight measurements at week 5 was unattainable, thus creating a gap in the continuity of the data presented. Consequently, this study pivoted to deliver insights primarily based on the available data at the specified intervals of weeks 3 and 8. At week 8 post-euthanisation, intestinal contents were collected from the cecum, colon, and duodenum of 24 animals for 16S rRNA gene amplicon analysis and shotgun sequencing. For the gut histomorphology analysis, ileum tissues were sampled and preserved in formalin for storage and transportation.

### DNA extraction, amplification, and sequencing

DNA from centrifuged intestinal content and faecal samples was prepared for 16S rRNA sequencing. Pellets were transferred to tubes with 0.2-g glass beads (0.1 mm diameter) and 0.7-ml lysis buffer (500 mM NaCl, 50 mM ethylenediaminetetraacetic acid (EDTA), 50 mM Tris–HCl, pH 8, 4% sodium dodecyl sulphate (SDS)). After homogenising using Precellys 24 homogeniser (Bertin Technologies, Montigny-le-Bretonneux, France) for 55 s, samples were incubated at 85 °C for 15 min, with intermittent vortexing.

After centrifugation at 16,000 rcf for 5 min, 0.4-ml of the supernatant was transferred to 1.5-ml tubes containing 500-µl of binding buffer (5 M Gu-HCl, 30% isopropanol). After a brief vortex, it was centrifuged, and the supernatant was shifted to a DNA spin column (Enzymax LLC, Cat# EZC101, Lexington, KY). The column was washed twice with 800 µl of wash buffer (10 mM Tris–HCl, 80% ethanol, pH 7.5) and dried by centrifugation. DNA was then eluted with 50 µl of elution buffer (10 mM Tris–HCl) and analysed using a NanoDrop spectrophotometer (Thermo Fisher, Waltham, MA, USA) for concentration and quality.

For amplicon sequencing of the V3-V4 region of the 16S rRNA gene, dual index primer pairs (forward primer pro341F (5′-CCTACGGGNBGCASCAG-3′) and reverse primer pro805R (5′-GACTACNVGGGTATCTAATCC-3′)) with index, heterogeneity spacer, and Illumina sequencing linkers were used (Fadrosh et al. 2014). The 16S rRNA amplicon library was purified using AMPure XP Kits (Beckman Coulter, Brea, CA, USA) and sequenced on the Illumina MiSeq platform (2 × 250 bp paired-end). A minimum Phred score of 25 across 200 nt of the forward read was applied for downstream analysis.

The raw DNA sequences were demultiplexed using Cutadapt (Martin 2011) and analysed using Quantitative Insights into Microbial Ecology 2 (QIIME2) for microbiota analysis (Bolyen et al. 2019). Quality filtering, denoising, and chimaera removal were performed using the Dada2 plugin (Callahan et al. 2016) with recommended parameters. Taxonomy assignment was conducted using the SILVA v138.1 database (Quast et al. 2013) as the reference.

### Data analysis

Data analysis was carried out at the amplicon sequence variant (ASV) and genus levels, with a minimum of 2000 rarefied sequences per sample, chosen after extensive exploratory analysis. Various levels of sequences, ranging from 1000 to 5000, were evaluated to understand the impact on the robustness of the dataset and the representation of microbial diversity. The level of 2000 sequences was found to be optimal as it allowed for the inclusion of a maximum number of valid samples without compromising the reliability and robustness of the dataset.

Downstream analysis and visualisation were performed using R packages Phyloseq (McMurdie and Holmes 2013), Vegan (Dixon 2003), and Microeco (Liu et al. 2021).

### Metagenomic sequencing and analysis

Metagenomic data for the study were obtained through the shotgun sequencing of genomic DNA from cecal samples obtained at the end of the trial at week 8. We specifically selected cecal samples for metagenomic analysis due to the cecum’s role as a major site of microbial fermentation and its high microbial abundance and diversity (Ley et al. 2008; Donaldson et al. 2016). The richness of microbiota in the cecum provides a comprehensive overview of microbial functions and pathways, allowing a detailed exploration of host-microbiome interactions.

DNA quality and quantity were assessed using a NanoDrop Qubit 3.0 (Thermo Fisher, Waltham, MA, USA) and agarose gel electrophoresis. DNA was fragmented using ultrasonication with a Covaris S220 (Covaris, Woburn, MA, USA) followed by end repair, dA-tailing, adapter ligation, and purification. After purification, the DNA was size-selected, and PCR amplified to construct a library. The concentration and insert size of the diluted library were determined using the Agilent 2100 (Agilent, Santa Clara, CA, USA). To ensure accurate sample concentrations and reliable sequencing data, the effective concentration of each library in the library mix was determined prior to sequencing using qPCR. The sequencing process was performed using an Illumina Novaseq6000 platform with a 150PE configuration.

After sequencing, the integrity of the data was verified via the generation of a cryptographic hash utilising the Message Digest Algorithm 5 (Rivest 1992). Initial quality control of the sequence data and removal of the sequencing adaptors were carried out with fastp v0.20.0 (Chen et al. 2018). This was followed by further processing and quality control measures implemented using KneadData v0.7.10, involving the utilisation of Trimmomatic (Bolger et al. 2014) for the filtering and trimming of quality, set at leading and trailing base quality of three and a sliding window threshold of 4:15. The host-DNA contamination was removed by aligning the sequences with pig reference genome Sscrofa11.1 (RefSeq GCF_000003025.6) in NCBI database after indexing.

This process yielded a total of around 822 million quality-filtered, host-DNA decontaminated sequences. Per sample, the sequence counts ranged from 20.7 million sequences minimum to 44.1 million sequences maximum, with an average of around 35.73 ± 4.96 million (mean ± SD) sequences with a minimum Phred score of 35. Before quality control, there were around 945 million sequences in total, with 41.10 ± 2.91 million (mean ± SD) sequences per sample, ranging from 20.7 million sequences minimum to 44.1 million maximum sequences per sample.

The cleaned and quality-filtered and decontaminated sequences were subsequently analysed utilising the computational tool HUMAnN v3.6 (Abubucker et al. 2012) using UniRef90 databases (Suzek et al. 2007) to analyse molecular functions and metabolic pathways.

The derived functional data were examined and visualised using the R programming language with a range of packages and tools including Phyloseq (McMurdie and Holmes 2013), Vegan (Dixon 2003), and Microeco (Liu et al. 2021). Lastly, statistical analyses and plotting were performed using GraphPad Prism v9 (GraphPad Software, San Diego, CA, USA) and Primer-e V7 (Primer-E Ltd, Auckland, New Zealand).

### Histology

For histomorphology analysis, ileum samples were fixed in a 10% neutral buffered formalin and then embedded in paraffin wax. The tissue processing involved several steps, including fixation, paraffin embedding, and microtoming. The embedded samples were cut into thin sections of 4-µm thickness using Leica RM2135 microtome (Leica Biosystems, Nussloch, Wetzlar, Germany). These sections were stained with haematoxylin and eosin (H&E) (POCD, North Rocks, NSW, Australia). The slides were scanned using a Nikon Eclipse Ci-L Plus microscope (Nikon Corporation, Tokyo, Japan) and Panoptiq™ software (ViewsIQ Inc., Vancouver, Canada). Measurements of villus height, crypt depth, villus area, and the number of goblet cells were performed on ten randomly selected well-positioned villi per slide, with a total of 12 slides analysed for each group.

### Statistical materials and methods

Statistical analysis was conducted using GraphPad Prism 9 (GraphPad Software, Boston, MA, USA) to compare animal weights, alpha diversity, histological measurements, and univariate analysis. To evaluate alpha diversity, we calculated the count of ASVs (amplicon sequence variants) present in each sample, as well as the Chao1, Shannon, and Simpson indices. These indices take into account both the evenness and richness of species within a given sample, providing a comprehensive measure of diversity. Univariate analyses were performed in Primer-e v7 (Anderson et al. 2008), and the results were visualised using GraphPad Prism 9. To assess beta diversity, we employed the UniFrac distance metric, which considers the phylogenetic relationships between ASVs to measure dissimilarity among treatment groups. Both unweighted and weighted UniFrac distances were used to capture the presence/absence and abundance of ASVs, respectively. To evaluate the significance of differences between groups, UniFrac distances were used to perform a pairwise permutational multivariate analysis of variance (PERMANOVA) with 999 random permutations and similarity percentage (SIMPER) using Primer-e v7 (Anderson et al. 2008).

## Results

### Animal health and performance

**Fig. 1:**
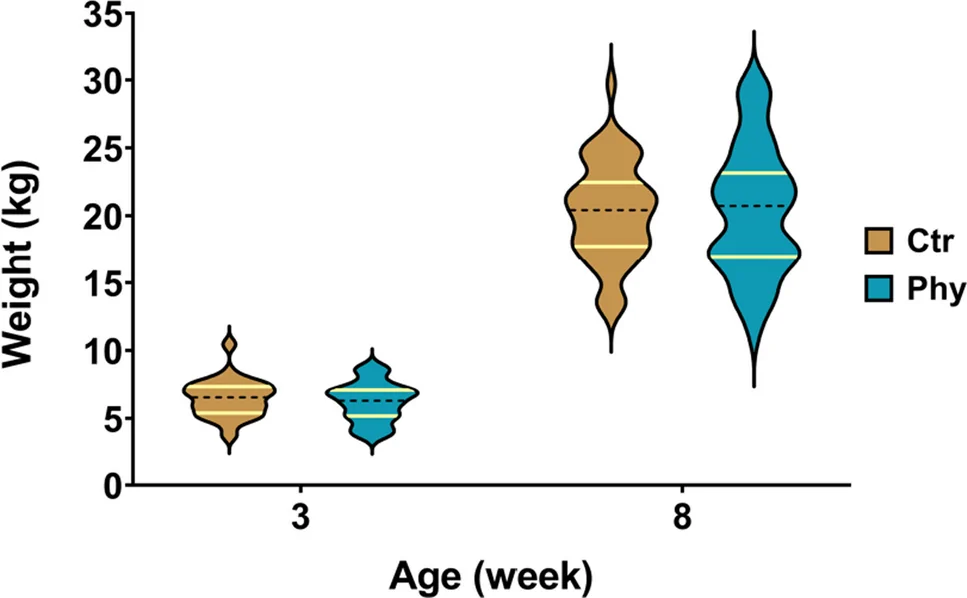
Violin plot representing the weight distributions for the Ctr and Phy groups at weeks 3 and 8. Both the 25th and 75th percentiles are visibly marked with yellow lines on each plot to illustrate the data distribution more explicitly. Specifically, at week 8, the 75th percentile for the Ctr group is 22.46 and for the Phy group 23.15. The number of animals used was as follows: week 3, Ctr, *n* = 50; Phy, *n* = 50; and week 8, Ctr, *n* = 48; Phy, *n* = 44

The effect of PFA on the growth performance of weaner pigs during the grower period was evaluated (Fig. 1, and Supplementary Table S1). At the commencement of the trial, the mean body weights (BW) of the control and treatment groups were comparable (6.486 kg and 6.218 kg, respectively, *p* = 0.348). Similarly, there was no statistically significant difference (*p* = 0.913) in average body weight between the control and treatment groups at week 8. However, after the 5-week trial period, animals in the treatment group demonstrated higher and wider distribution above the 75th percentile compared to the control group.

### Overall microbiota composition

**Fig. 2:**
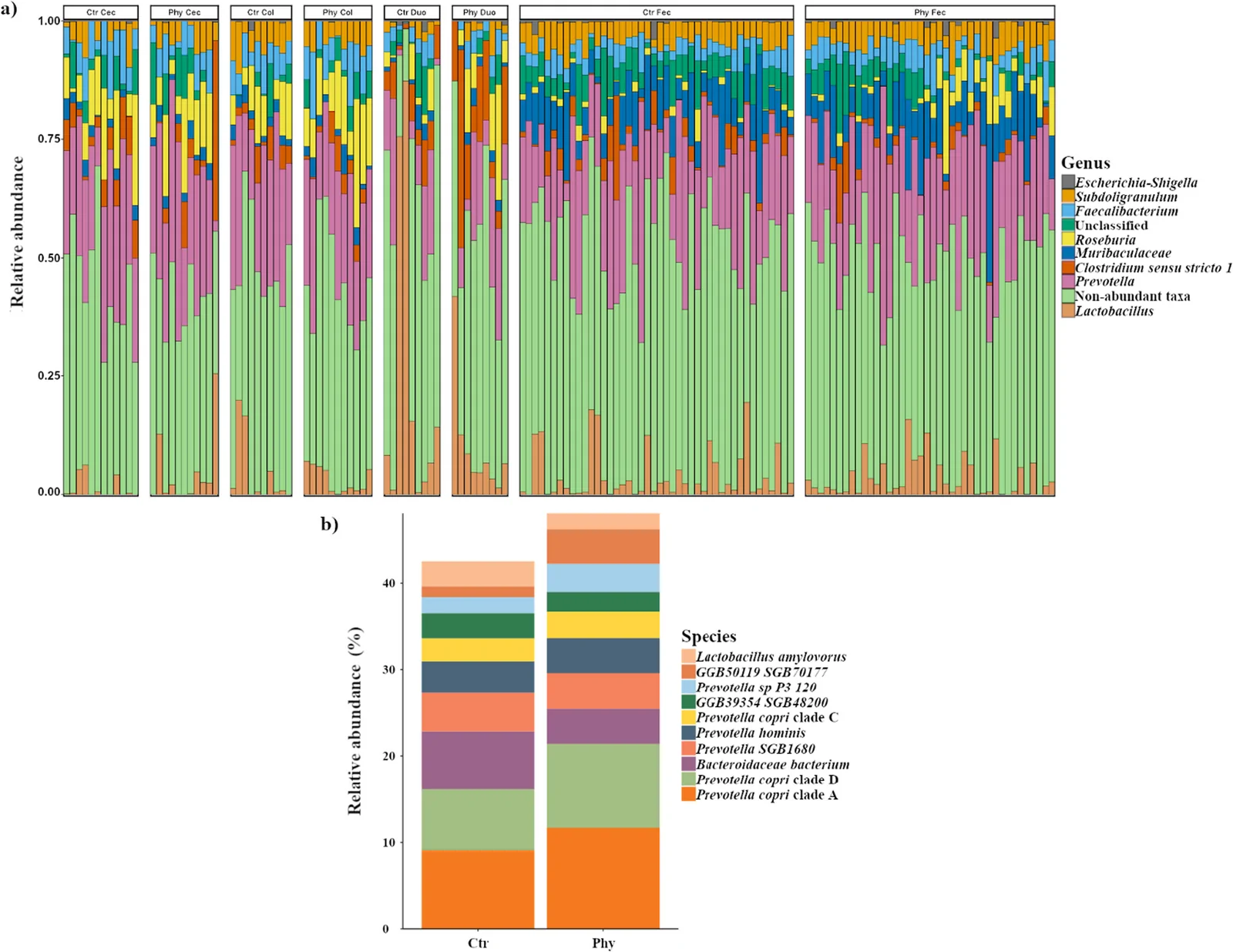
Microbial community composition in different gut sections at week 8. **a** Relative abundance of the top ten most prevalent genera between Ctr and Phy in all of the sampled gut sections (Duo = duodenum, Col = colon, Cec = cecum, Fec = faeces) in week 8. The respective number of animals used for each section is as follows: Cec (Ctr, *n* = 12; Phy, *n* = 11), Duo (Ctr, *n* = 9; Phy, *n* = 9), Col (Ctr, *n* = 10; Phy, *n* = 11), and Fec (Ctr, *n* = 44; Phy, *n* = 40). **b** Species-level relative abundance based on metagenomic sequencing between Ctr and Phy group at week 8 in the cecal samples (Ctr, *n* = 12; Phy, *n* = 11)

The relative abundance of the top ten most prevalent genera between control and treatment groups at the end of the trial is presented in Fig. 2a. Genera such as *Prevotella*, *Lactobacillus*, *Clostridium *sensu stricto* 1*, *Roseburia*, *Faecalibacterium*, *Subdoligranulum*, *Muribaculaceae*, and *Escherichia-Shigella* were consistently observed across all sampled gut sections. *Prevotella* remained the predominant genus across both groups in all gut sections, with *Roseburia* showing increased abundance in the treatment group.

For a deeper insight, we conducted a species-level analysis using metagenomic sequencing, focusing solely on the cecum samples. As illustrated in Fig. 2b, the findings at the species level were aligned with those observed at the genus level. Additionally, our metastat analysis of the species-level data from the cecum samples confirmed no significant variations between the control and treatment groups.

**Fig. 3:**
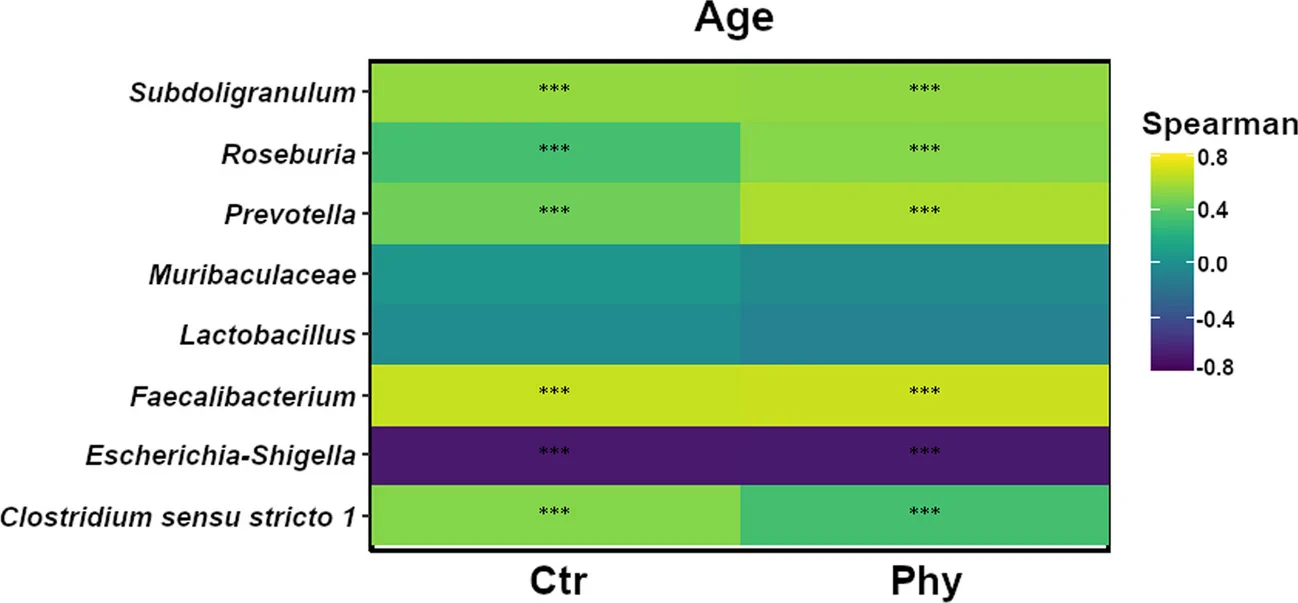
Correlation heatmap displaying the eight genera with the most notable temporal correlation patterns after week 8 of the trial period, reflecting changes from weeks 3 to 8. Age is correlated with changes in microbiota in both Ctr and Phy groups. Significance levels are marked with an asterisk: **p < 0.001, ***p < 0.0001

The correlation heatmap (Fig. 3) elucidates the relationship between the top eight taxa and the age of the pigs, specifically focusing on the changes observed from weeks 3 to 8, for both the control and treatment groups, using Spearman’s rank correlation coefficient. This method effectively details how the abundance of specific microbiota either increases or decreases with age in both the control and treatment groups. A higher coefficient with a significant *p*-value indicates a strong positive correlation with age, meaning that the abundance of the specific microbiota increases as pigs age.

In Fig. 3, *Roseburia* and *Prevotella* had higher Spearman’s rank correlation coefficients in the treatment group compared to the control group, indicating increasing abundance as the pigs aged. *Faecalibacterium* demonstrated increasing abundance with age in both control and treatment groups, as evidenced by higher correlation values in both groups. *Escherichia-Shigella* showed a negative correlation coefficient in both the control and treatment groups, suggesting a decrease in abundance as the pigs aged. *Clostridium *sensu stricto* 1* exhibited a positive temporal correlation in both groups, indicating increased abundance with age. These results emphasise the correlation between specific microbiota and age (weeks 3 to 8) in both groups, showing the temporal changes in gut microbiota composition in response to PFA-supplemented and non-PFA-supplemented animals.

### Alpha diversity

**Fig. 4:**
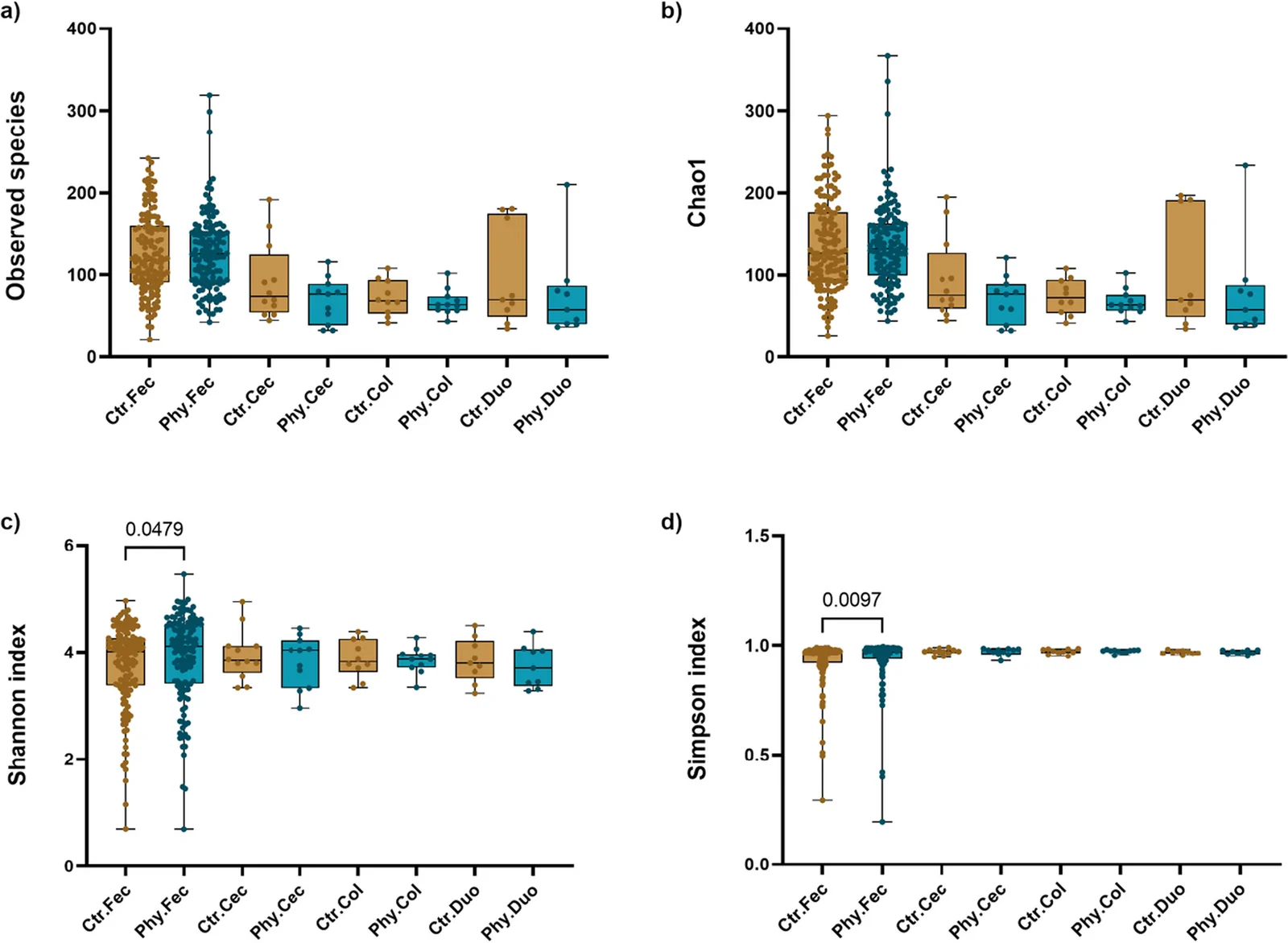
Comparison of alpha diversity measures across gut sections between Ctr and Phy groups. **a** Observed species, **b** Cho1, **c** Shannon index, and **d** Simpson index

Alpha diversity was assessed to evaluate the diversity and richness of gut microbiota species across faecal, caecal, colonic, and duodenal samples (Fig. 4). While there were no significant differences noted in the observed species and Chao1 index metrics with the exception of faecal samples, the control group demonstrated higher index values in the cecum, colon, and duodenum compared to the treatment group, indicating possible higher richness and diversity in the microbiota profile. This observation could benefit from an increase in sample size. In faecal samples, the treatment group demonstrated significantly higher values for both Shannon and Simpson indices (*p* = 0.0479 and *p* = 0.0097, respectively). Notably, for the Simpson index, we employed the diversity version (‘1-λ′’), where values closer to 1 indicate greater species diversity. The results suggest an enhanced species diversity of gut microbiota in the treatment group.

### Faecal microbiota composition changes over time

**Fig. 5:**
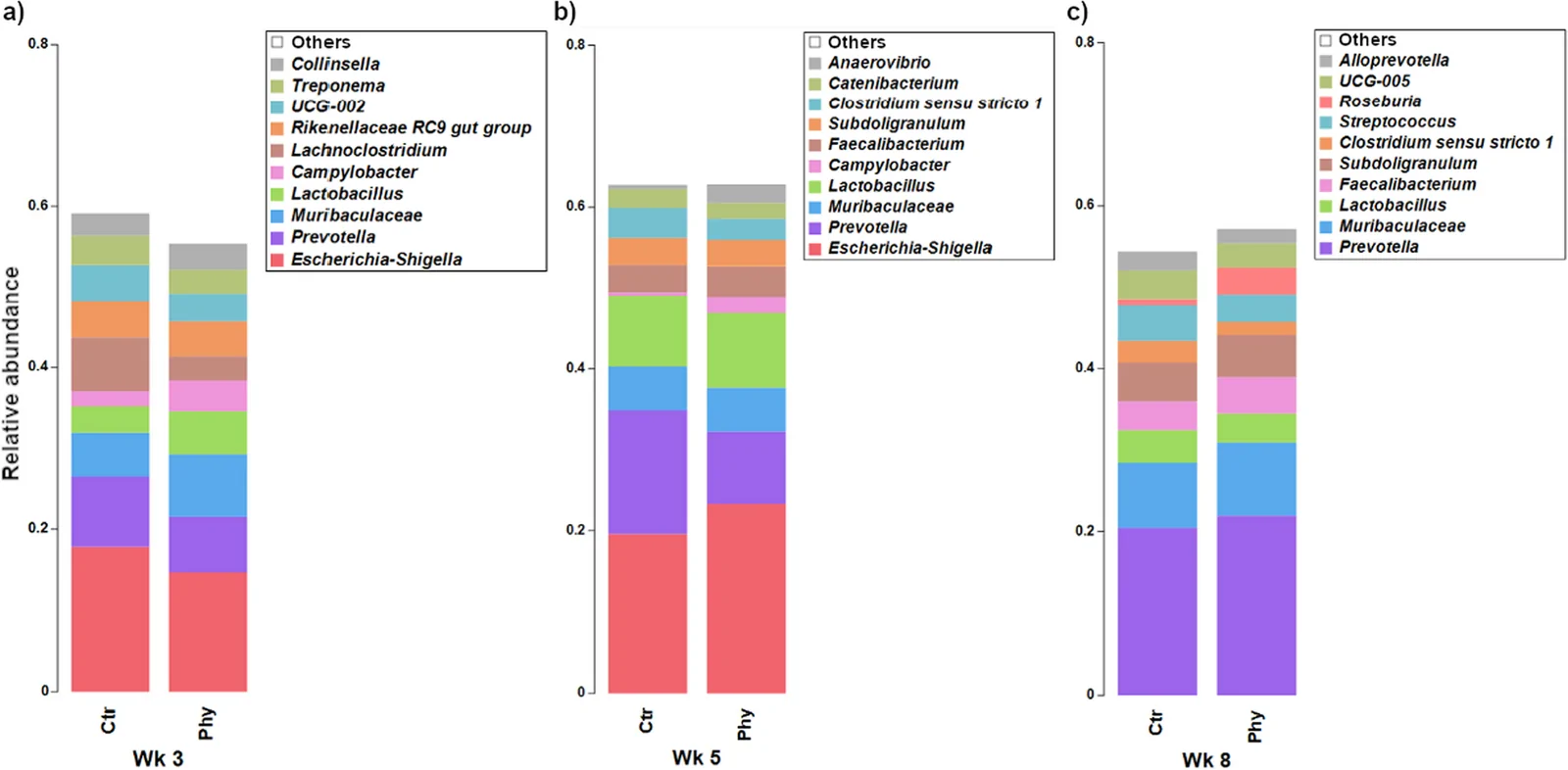
Stacked bar chart displaying the relative abundance of the top ten genera in faecal samples across three age groups. **a** Week 3, **b** week 5, and **c** week 8

A relative abundance stacked bar chart was created to evaluate the microbial genera in faecal samples across three distinct age groups (weeks 3, 5, and 8). In Fig. 5, the stacked bar chart provides insight into the changes in the relative abundance of microbial genera over time between the two groups. The most abundant genus in both groups at weeks 3 and 5 was *Escherichia-Shigella*; however, at week 8, the most abundant genus shifted to *Prevotella*, and *Escherichia-Shigella* was no longer present in the top ten most abundant genera. *Prevotella* was highly abundant in the control group in week 5 compared to the treatment group, but in week 8, this genus was more abundant in the treatment group.

The similarity percentage (SIMPER) analysis was performed on faecal samples collected at week 8 to assess the contribution of each genus (%) to the dissimilarity between the two groups (Supplementary Table S2). The dissimilarity was calculated using the Bray–Curtis dissimilarity matrix. Based on the SIMPER analysis, *Prevotella* contributed the highest followed by *Muribaculaceae* and *Roseburia*, in the differences in microbial profile between the control and treatment groups. All these genera had higher abundances in the treatment group. Other important genera contributing to the differences were *Streptococcus*, *Clostridium *sensu stricto* 1*, and *UCG-005* with higher abundances in the control*.*

### Beta diversity

**Fig. 6:**
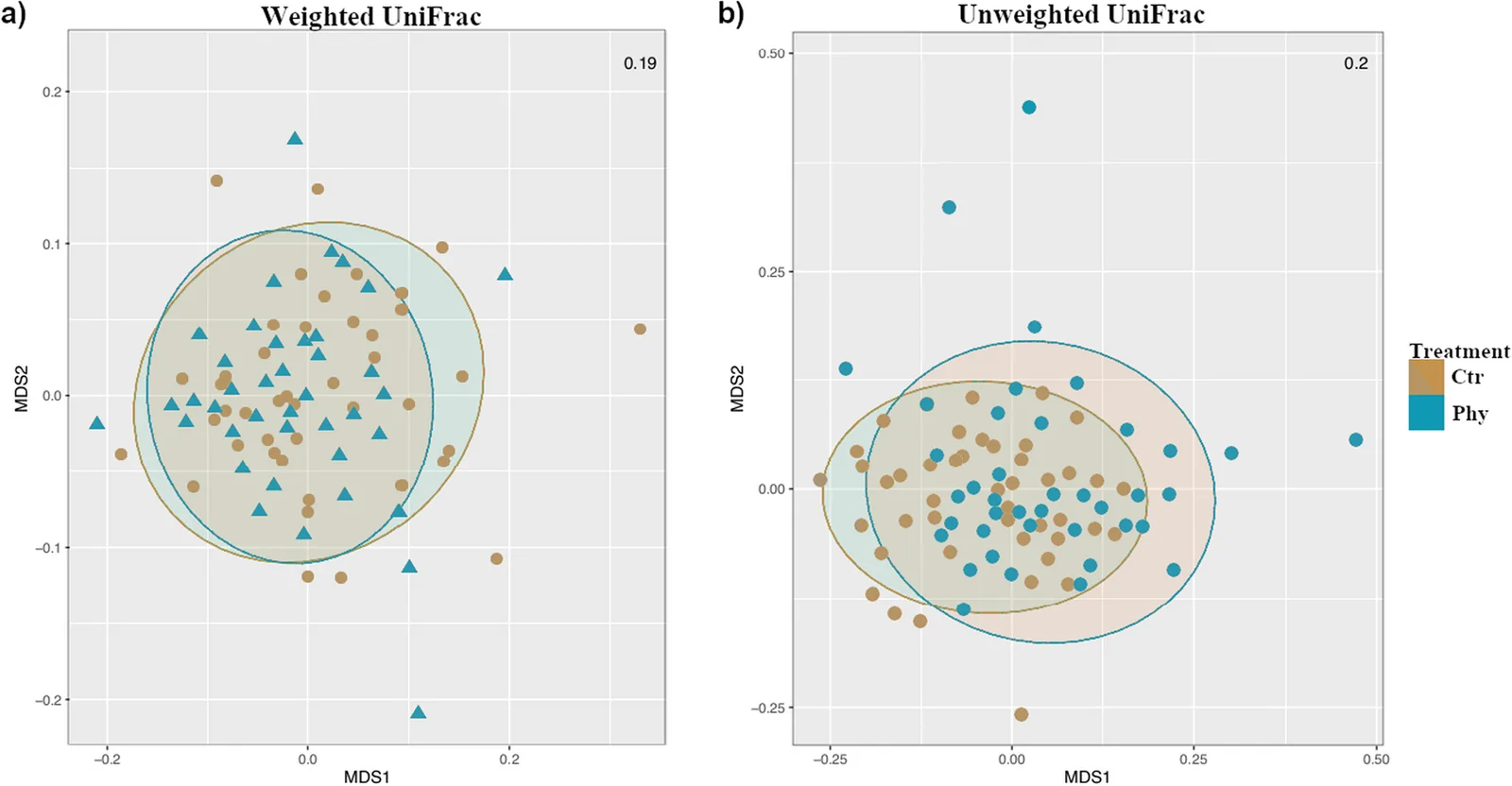
Non-parametric multidimensional scaling (NMDS) ordination of samples based on weighted (**a**) and unweighted (**b**) UniFrac distances at week 8. The stress value for NMDS is shown in the corresponding plots

The assessment of beta diversity was conducted by PERMANOVA and non-parametric multidimensional scaling (NMDS) of the weighted and unweighted UniFrac distances metrics (Table 1, and Fig. 6). These metrics consider both the presence or absence and the relative abundance of microbial taxa, enabling a detailed understanding of the similarities and dissimilarities in gut microbiota composition under the influence of various factors tested, including treatment, gender, age, and origin of samples.

**Table 1:** Summary of PERMANOVA analysis on faecal samples across different age groups to assess treatment effects. ‘*ns*’ denotes non-significant differences

Age (weeks)	Measure (UniFrac)	*p*-value	Significance
3	Weighted	0.787	ns
	Unweighted	0.316	ns
5	Weighted	0.061	ns
	Unweighted	0.095	ns
8	Weighted	0.119	ns
	Unweighted	0.001	***

***p < 0.0001

In unweighted UniFrac distance analyses, there were significant differences (*p* < 0.0001) in the gut microbiota composition between the two groups at week 8. The non-significant findings at weeks 3 and 5 of unweighted UniFrac analysis indicate no substantial differences in the overall microbial community structure between both groups during these time points.

The NMDS analysis plots were generated to assess the gut microbiota changes based on weighted and unweighted UniFrac distances between the control and treatment groups from the faecal samples at week 8 (Fig. 6). The NMDS plots illustrated contrasting points between the control and treatment groups in unweighted UniFrac analyses, indicating a degree of dissimilarity in the gut microbiota composition aligning with the PERMANOVA result. However, faecal microbiota did not show clear separation in microbial community composition for the weighted UniFrac distance.

### LEfSe analysis: identifying microbial biomarkers in pig gut microbiota

**Fig. 7:**
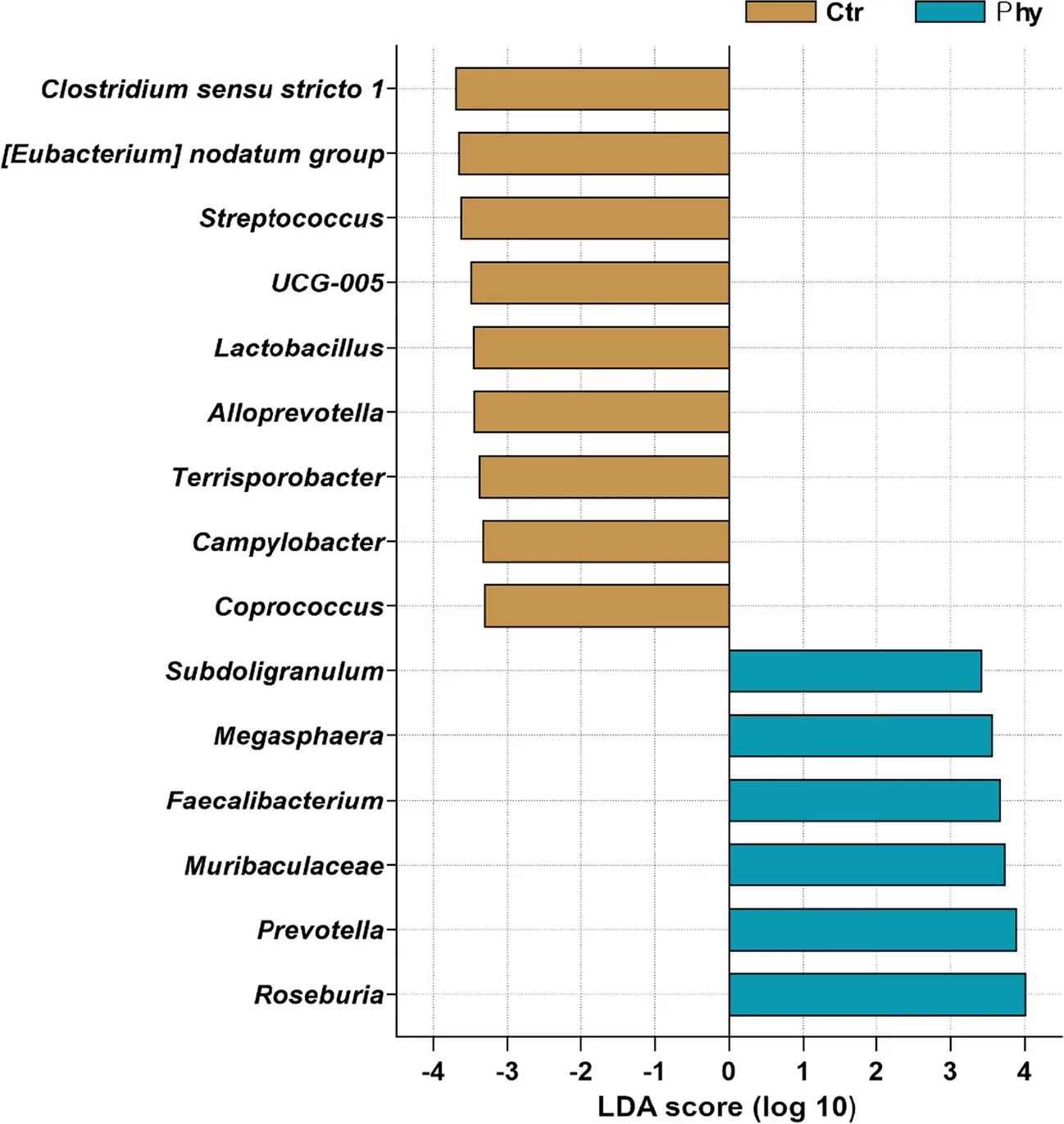
LEfSe histogram representing taxonomic biomarkers in the pig gut microbiota. The analysis was conducted using an LDA score threshold of > 3.3

The linear discriminant analysis effect size (Dethlefsen and Relman) histogram in Fig. 7 displays the bacterial taxa that showed the most significant differential abundance between the control and treatment groups. The LEfSe too uses non-parametric factorial Kruskal–Wallis (KW) sum-rank test and Wilcoxon rank-sum test followed by the linear discriminant analysis (LDA) to rank the differentially abundant taxa based on LDA in the control group. *Clostridium *sensu stricto* 1* had the highest LDA score, followed by *[Eubacterium] nodatum group*, *Streptococcus*, *UCG-005*, *Lactobacillus*, *Alloprevotella*, *Terrisporobacter*, *Campylobacter*, and *Coprococcus*. On the other hand, in the treatment group, the discriminative genera that stood out compared to the control group were *Roseburia*, *Prevotella*, *Muribaculaceae*, *Faecalibacterium*, *Megasphaera*, and *Subdoligranulum*.

### Sex-specific univariate analysis

In the experiment, we conducted a sex-specific univariate analysis to compare taxa abundance between the control and treatment groups, using faecal samples. In male piglets (Supplementary Fig. S1A), our analysis revealed that the control group had a higher abundance of the *[Eubacterium] nodatum group* and *Streptococcus* than the treatment group. In contrast, *Roseburia* was more abundant in the treatment group. In the female piglets (Supplementary Fig. S1B), we identified a higher abundance of *UCG-005* and *Alloprevotella* in the control group, compared to the treatment group. Meanwhile, *Faecalibacterium* was higher in abundance in the treatment group.

### Histological evaluation of ileal morphology

**Fig. 8:**
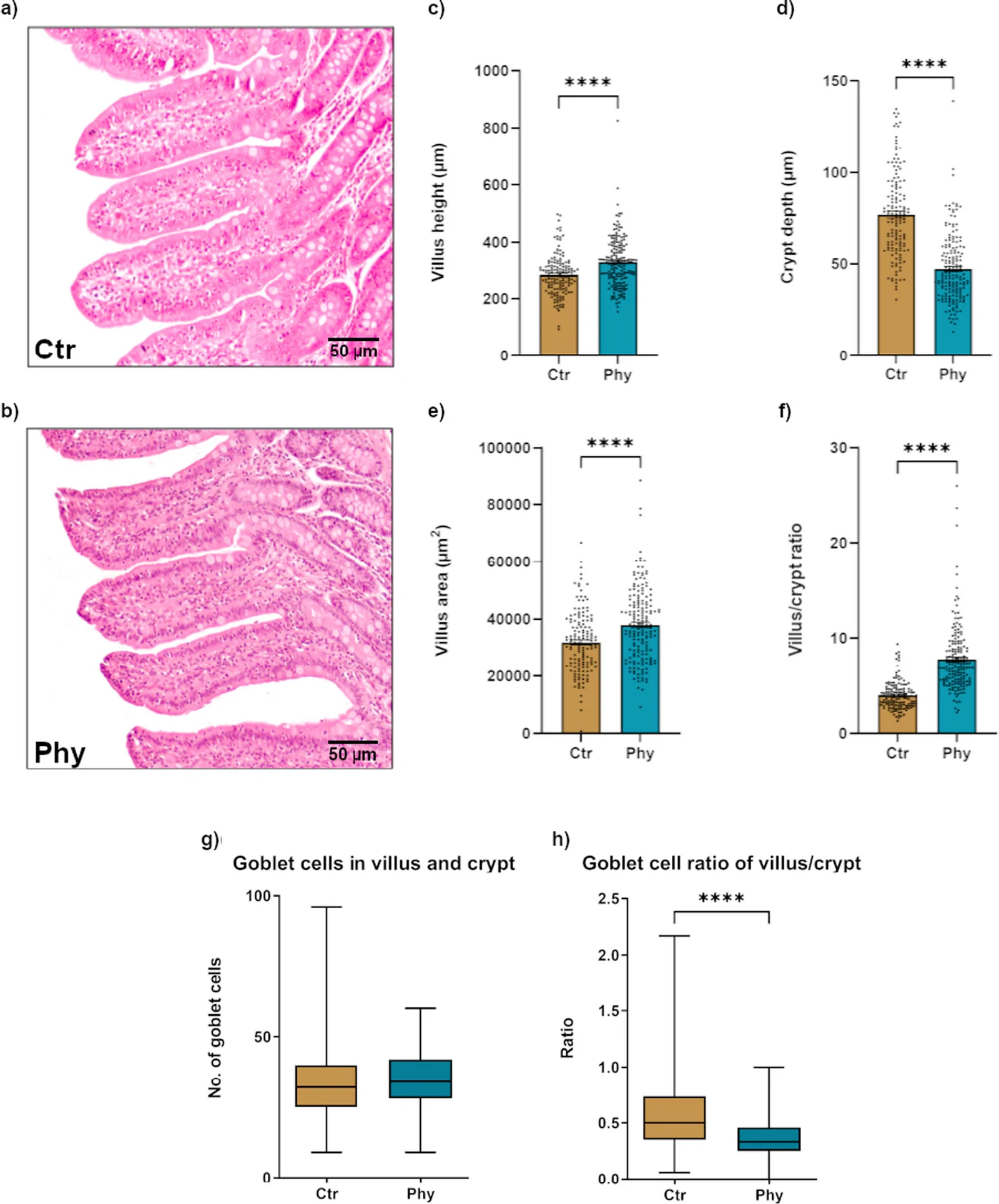
Comparative histological analysis of ileal tissue from Ctr and Phy groups. **a** Representative histology of ileal villi and crypt from the Ctr group. **b** Representative histology of ileal villi and crypt from the Phy group. Scale bar = 50 µm. **c** Villus height distribution across both groups. **d** Crypt depth distribution across both groups. **e** Villus area distribution across both groups. **f** Villus/crypt ratio across both groups. **g** Boxplot showing the number of goblet cells in villus and crypt for both groups. **h** Boxplot showing the ratio of villus/crypt goblet cells for both groups. Statistical significance is indicated with **** (*p* < 0.00001)

The histomorphology analysis looked at several parameters, including villus height (Fig. 8a), crypt depth (Fig. 8b), villus area (Fig. 8c), villus/crypt ratio (Fig. 8d), number of goblet cells in villus and crypt (Fig. 8e), and goblet cell ratio of villus/crypt (Fig. 8f), to gain insights into the changes in the structural organisation of the intestinal epithelium between the control and treatment group. In comparison to the control group, the treatment group exhibited notable alterations in the structural organisation of the intestinal epithelium. Specifically, the treatment group demonstrated higher values of villus height, villus area, and villus/crypt ratio, indicating increased dimensions and architectural complexity of the villi. Conversely, the crypt depth in the treatment group was substantially lower, suggesting a shallower depth of the intestinal crypts. There was no difference in the number of goblet cells along the villus/crypt axis between groups. However, in the treatment group, goblet cells were mainly located in the crypt epithelium and there was a statistically significant difference (*p* < 0.0001) in villus goblet cells/crypt goblet cells ratio (Fig. 8).

### Metabolic pathway analysis

**Fig. 9:**
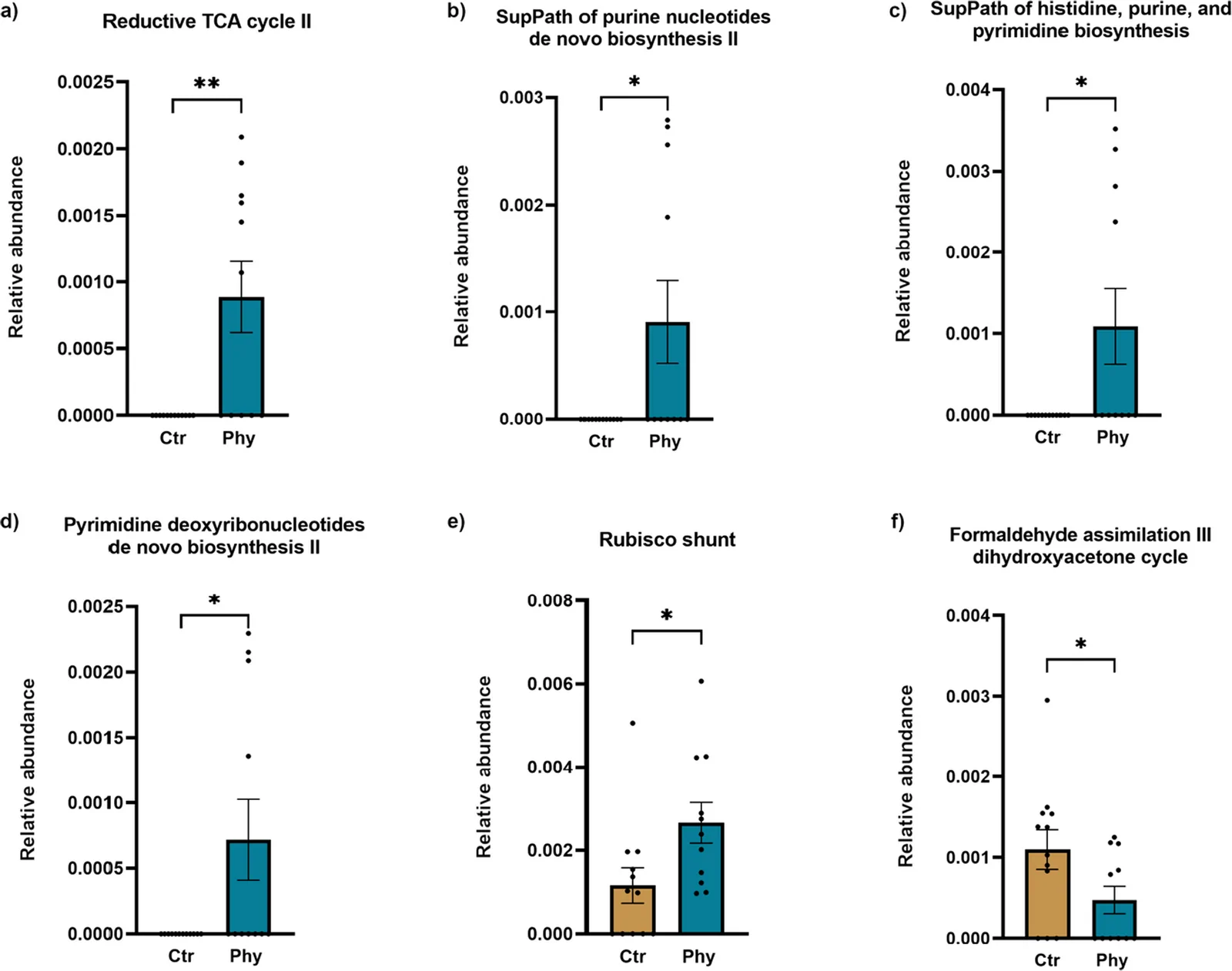
Differential abundance of metabolic pathways between Ctr and Phy groups as revealed by metagenomic analysis. **a** Reductive TCA cycle II; **b** superpathway of purine nucleotides de novo biosynthesis II; **c** superpathway of histidine, purine, and pyrimidine biosynthesis; **d** pyrimidine deoxyribonucleotides de novo biosynthesis II; **e** Rubisco shunt; and **f** formaldehyde assimilation III dihydroxyacetone cycle pathway. Statistical significance is indicated by asterisks where *p < 0.05 and **p < 0.001

In our examination of metabolic pathways, several significant differences were observed between the control and treatment groups (Fig. 9). Statistically significant pathways that showed higher abundance in the PFA-supplemented group included PWY-5392 (reductive tricarboxylic acid (TCA) cycle II), DENOVOPURINE2 PWY (superpathway of purine nucleotides de novo biosynthesis II), PRPP PWY (superpathway of histidine, purine, and pyrimidine biosynthesis), PWY-7187 (pyrimidine deoxyribonucleotides de novo biosynthesis II), and PWY-5723 (Rubisco shunt). In contrast, the control group demonstrated an elevated abundance of the P185-PWY (formaldehyde assimilation III dihydroxyacetone cycle).

Additional pathways were identified with noticeable but not statically significant shifts in abundance between the two groups (Supplementary Fig. S2). The control group showed a slightly higher abundance of the PWY-3781 (aerobic respiration I (cytochrome c)) and PWY-5505 (L-glutamate and L-glutamine biosynthesis). On the other hand, the treatment group demonstrated an increase in the RUMP PWY (formaldehyde oxidation I), PWY-5028 (L-histidine degradation II), and PWY-6328 (L-lysine degradation X). These results underscore the potential impact of PFAs on the metabolic functionality within the pig gut microbiota.

## Discussion

In this study, we explored the influence of PFA on the gut microbiota, metabolic pathways, gut histomorphology, and overall performance of weaned piglets. We aimed to investigate how early intervention with PFA could shape the gut microbiota composition and subsequently influence the health and growth of the pigs at the weaning phase. This stage has been numerously reported as associated with intestinal inflammation and systemic proinflammatory response, which are risk factors increasing the incidence of digestive disorders, where bacterial diseases usually play a major role (McCracken et al. 1999). Therefore, by manipulating the pigs’ gut environment using PFA, we aimed to achieve a more favourable microbial balance and enhance their well-being.

Beta diversity analysis was employed to investigate the overall composition of the gut microbiota in both the control and treatment groups at three time points: weeks 3, 5, and 8. Notably, the unweighted UniFrac distance measurement indicated a significant divergence (*p* < 0.0001) in the microbial community structures between the two groups at week 8. The distinct microbial profiles for each group at this age are potentially linked to variations in the abundance or prevalence of certain key taxa. These variations likely contribute to the measurable divergence in gut microbiota composition, suggesting that the introduction of PFA may have influenced the presence or absence of specific microbial species in the gut microbiota of the weaned piglets over time. However, our PERMANOVA analysis found no significant differences at the earlier time points of weeks 3 and 5. While this lack of statistical significance does not conclusively demonstrate the absence of an effect of PFA on microbial abundance or proportional representation during these periods, it does indicate that any such effects were not severely altering overall microbial structure but were instead more targeted at specific taxa.

Alpha diversity was higher in the treatment group as measured by the Shannon and Simpson indices in the faeces. The higher Shannon index in the treatment group points to a more diverse and evenly spread microbial community. Using the Simpson index (‘1-λ′’), where a value nearing 1 represents higher species diversity, our findings also demonstrated increased diversity in the treatment group. It is widely accepted that enhanced microbial diversity is typically advantageous for the host. For instance, there has been an observed inverse correlation between the richness of the gut microbiome and triglyceride (TG) concentrations, suggesting that increased microbial diversity correlates with reduced TG levels (Fu et al. 2015). Additionally, a positive relationship between the diversity of the gut microbiome and high-density lipoprotein cholesterol (HDL-C) quantities has been documented, illustrating the potential benefits of a diverse microbial community (Matey-Hernandez et al. 2018).

The usage of antibiotics has been shown to exert an unfavourable impact on gut microbiota diversity (Ubeda and Pamer 2012) causing a noticeable reduction in the faecal microbiota’s diversity (Dethlefsen and Relman 2011). This reduction of diversity can destabilise the equilibrium of the gut ecosystem, promoting the proliferation of select opportunistic pathogens at the loss of advantageous commensals (Lozupone et al. 2012). The decline in microbial diversity is intrinsically tied to adverse health outcomes. Specifically, a gut microbiota with less diversity might weaken the host’s resilience to the invasion of the mucosal surfaces by external pathogens, resulting in increased susceptibility to infections in the gut (Buffie and Pamer 2013; Keesing and Ostfeld 2015). In addition, a less heterogeneous microbiota could alter metabolic functions, including nutrient uptake and energy regulation, which are crucial roles assumed by the microbiota (Holmes et al. 2012). In contrast to antibiotics, it is noteworthy that the application of PFAs, as highlighted in this study, enhanced the preservation of the equilibrium and diversity of the microbiota in the faeces. The PFAs have been shown to maintain a robust and diverse microbial community, thereby allowing the beneficial commensals to thrive and contributing to a healthier host (Applegate et al. 2010; Windisch et al. 2008).

Despite initial lower weights, the treatment group demonstrated slight but non-significant improvements in average weight gain by week 8, although increased variability was observed within the group. These phytogenic supplements are designed and optimised to enhance palatability and promote digestibility by stimulating enzyme secretion in the gut (Mohana Devi et al. 2015). Consequently, several studies observed corresponding improvements in growth performance (Chang et al. 2023; Mohana Devi et al. 2015).

The capacity of PFAs to enhance piglet growth performance is not an isolated effect. This enhancement can be traced back to alterations in the gut microbiota, as revealed by our 16S rRNA gene amplicon analysis, which showed a noticeable shift in specific microbial taxa in the animals treated with PFAs. Notably, the abundance of beneficial genera *Roseburia*, *Faecalibacterium*, and *Prevotella* was significantly increased in the treatment group, indicating the beneficial effects of PFA treatment. These specific bacteria have the ability to break down various complex carbohydrates and produce significant amounts of short-chain fatty acids (SCFAs) (Dou et al. 2017). SCFAs, particularly butyrate, play a crucial role in maintaining gut health (Louis and Flint 2009; Rios-Covian et al. 2016). Furthermore, these bacteria are known to protect against *Escherichia coli*-induced intestinal infections (Singh et al. 2015), and their early presence in the GIT may equip piglets with a greater ability to digest glycans (Guevarra et al. 2018), preparing them for a post-weaning diet full of complex carbohydrates. This indicates a mature-like microbiota that assists healthy pigs during their dietary transition in the weaning period (Chen et al. 2017).

Conversely, we observed a marked reduction of potentially pathogenic bacteria such as *Clostridium *sensu stricto* 1* and *Campylobacter*. Many species within these genera have been associated with conditions such as food poisoning, enteritis, and other digestive disorders (Allos 2001; Rupnik et al. 2009). Their decrease in the PFA-fed piglets may indicate a healthier, more balanced gut microbial environment, reducing susceptibility to infections and gut disorders.

A shift in the gut microbiota was also noted in the piglets’ transition from suckling to weaning. It has been reported that as piglets mature, the genus *Prevotella* progressively becomes more prevalent within their faecal microbial communities (Alain et al. 2014). *Prevotella* plays a significant role in adult animals’ GIT, contributing significantly to the breakdown of starch, plant polysaccharides, and mucoproteins (Ivarsson et al. 2014). Research by Frese et al. (2015) revealed a notable increase in *Prevotella* abundance from suckling to weaning (from 0.3% on average to 14.8%). This observation aligns with our findings, supporting that increased *Prevotella* abundance correlates with improved digestion and nutrient utilisation in weaned piglets as they transition from milk to solid feed diets.

In addition to our initial genus-level insights, we extended our analysis to the species level using metagenomic sequencing, concentrating on cecum samples due to their significant influence on microbiota composition and functionality (Donaldson et al. 2016). The findings from this species-level investigation (as illustrated in Fig. 2b) corroborated the patterns observed in our caecal 16S rRNA genus-level analysis (Fig. 2a), underlining the consistency across different taxonomic resolutions.

Our histological evaluation of ileal morphology pointed to significant differences between the control and treatment groups. We considered several parameters, including villus height, crypt depth, villus area, and villus/crypt ratio, to assess the structural organisation of the intestinal epithelium. Interestingly, the animals that received PFAs demonstrated greater values of these parameters compared to the control group. This suggests increased dimensions and architectural complexity of the villi, indicative of enhanced nutrient absorption.

The increased villus height and area are directly correlated with the intestine’s nutrient absorption capabilities due to their contribution to the surface area available for absorption (Montagne et al. 2003; Sklan et al. 2003). This implies a greater potential for nutrient utilisation and growth within this group. Simultaneously, a balanced crypt depth, a parameter indicative of cell turnover in the intestinal epithelium, was observed in the treatment group, suggesting a healthier gut environment by reflecting lower rates of cell replacement due to damage or loss (Pluske et al. 1997). In addition, an increased villus/crypt ratio, representing a mature and efficient absorptive surface relative to the cell production site, was seen in the treatment group, a promising indication of an optimal gut condition and improved gut structure and function, hence providing a more substantial barrier against potential pathogens (Sakamoto et al. 2000).

As part of the epithelial lining of the intestine, goblet cells are responsible for the production and maintenance of the protective mucus layer, which acts as the first line of defence against pathogens and harmful substances (Johansson and Hansson 2016). Their primary function is the secretion of mucins, the major component of the mucus layer, which, in a well-hydrated state, facilitates the smooth passage of intestinal contents while providing a barrier against potential pathogens (Pelaseyed et al. 2014). Remarkably, our findings indicate that in the treatment group, goblet cells were primarily localised within the crypts. This distribution may have substantial implications for the protective capacity of the gut under the stresses of weaning. It is within the crypts where the renewal of the intestinal epithelium occurs. Here, the stem cells divide and differentiate into various cell types, including goblet cells, which migrate upwards along the crypt-villus axis (Barker 2014). Therefore, having a higher concentration of goblet cells in the crypts could mean an enriched source of new cells ready to replenish the mucosal barrier, potentially enhancing the resilience of the gut in response to weaning stress (Kim and Ho 2010).

The alterations in the intestinal morphology we observed align closely with previous studies, such as Michiels et al. (2010), who found a heightened ratio of villus height to crypt depth in the distal small intestine of piglets treated with PFAs. This morphological enhancement could feasibly be the foundation of the improved growth performance. Intriguingly, the beneficial effects of PFAs on intestinal morphology are not restricted to swine; similar findings have been reported in poultry. Both Demir et al. (2003) and Jamroz et al. (2006) have reported improved gut morphology in chickens upon supplementing their diets with PFAs.

These morphological changes might not occur independently but could represent part of a more extensive biological response including alterations in the activity of metabolic pathways within the gut microbiota. By profiling these metabolic pathways, we aim to unravel the potentially complex mechanisms behind the beneficial effects of PFA supplementation.

Notably, we found that the administration of PFAs led to specific metabolic alterations within the gut microbiota of piglets. The enhancement of nucleotide biosynthesis pathways, particularly the superpathway of purine nucleotides de novo biosynthesis II (DENOVOPURINE2 PWY) and the superpathway of histidine, purine, and pyrimidine biosynthesis (PRPP PWY), may reflect the need for increased nucleic acid synthesis in response to PFA supplementation. PFAs, as a mixture of herbs, spices, and derived products, have been suggested to affect multiple biological processes that could influence nucleotide turnover, such as enhancing growth, modulating immunity, and impacting intestinal morphology (Windisch et al. 2008). These nucleotide-related pathways could also be linked to the improved gut morphology observed in PFA-supplemented piglets. Enhanced intestinal crypt cell proliferation, which requires ample nucleotide supply for DNA replication, might be one possible mechanism underlying this observation (Moeser et al. 2017).

Aerobic respiration I (cytochrome c) (PWY-3781) was slightly less abundant in PFA-supplemented piglets. While this pathway is fundamental for energy generation through oxidative phosphorylation (Mitchell 2011), its downregulation may reflect a shift towards alternative energy-generating pathways. Some of the compounds found in these additives, such as flavonoids and phenolic acids, may directly influence metabolic processes (Placha et al. 2014; Rajput et al. 2013; Yu et al. 2022). Studies have indicated that they can improve nutrient digestibility, leading to changes in metabolic rates. For example, a study by Rajput et al. (2013) demonstrated that dietary supplementation with PFAs improved the energy balance of broilers. These compounds may also influence the activity of key metabolic enzymes and modify the gut microbiota, leading to changes in the metabolic profile.

The L-glutamate and L-glutamine biosynthesis pathway (PWY-5505) was slightly less abundant in PFA-treated piglets. Glutamate and glutamine are essential for numerous cellular functions, including protein synthesis, pH homeostasis, and energy production (Newsholme et al. 2003). The decreased abundance of this pathway may be an adaptive response to altered dietary nutrient availability caused by PFA supplementation.

The formaldehyde oxidation I (RUMP PWY) pathway was more prevalent in the PFA group. Formaldehyde is a toxic metabolic intermediate that is produced in organisms as a part of various metabolic processes, and its detoxification is critical for cellular health (Grafstrom et al. 1983). An upregulation of this pathway could point towards an increased capacity of microbiota for formaldehyde detoxification in PFA-treated pigs, indicating a better adaptation to metabolise formaldehyde into less toxic compounds.

The L-histidine degradation II (PWY-5028) pathway and the L-lysine degradation X (PWY-6328) pathway, both involved in amino acid metabolism, were also found to be increased in the PFA group. Amino acids are the building blocks of proteins and can serve as energy sources (Wu 2013). The upregulation of these pathways might suggest an increased protein turnover or a shift in the metabolic strategies of the gut microbiota under PFA supplementation.

Our research highlights the potential of PFAs in influencing the gut microbiota in weaned piglets, particularly in faecal samples. It suggests that PFAs may enhance microbial diversity, influence specific metabolic pathways such as the superpathway of purine nucleotides de novo biosynthesis II and the formaldehyde oxidation I pathway, and impact the structural organisation of the intestinal epithelium. These changes not only indicate possible improvements in gut health but also potentially increase resilience to weaning stress, contributing to the overall well-being of piglets.

In understanding the efficacy of commercially available PFA blend, this study remained rooted in real-world applications. We acknowledged the blend’s inherent complexities and synergistic effects, aiming to provide insights immediately applicable to farmers and industry professionals. We recognise the value of studying the individual effects of each component within the blend in future research efforts.

Furthermore, the insights gained here position PFAs as a compelling alternative to antibiotics, avoiding problems associated with antibiotic use. This study sheds light on how early dietary interventions might optimise livestock health. Future research should delve deeper into the long-term implications of PFAs on livestock health and productivity, aiming to develop practical guidelines for their use in farming.

## Supplementary Information

Below is the link to the electronic supplementary material.

Supplementary file1 (PDF 353 KB)

## Data Availability

The raw sequencing files of the microbiota are available in the NCBI SRA database under the project number https://www.ncbi.nlm.nih.gov/bioproject/PRJNA997754.
