# Supplementary material for: Pioneering gut health improvements in piglets with phytogenic feed additives
Source: Appl Microbiol Biotechnol. 2024 Jan 17;108:142. doi: 10.1007/s00253-023-12925-2 (PMC10794284; doi:10.1007/s00253-023-12925-2)
Supplement: Supplementary file 1 [file 253_2023_12925_MOESM1_ESM.pdf]

Applied Microbiology and Biotechnology

Supplementary Material

Pioneering gut health improvements in piglets with phytogenic feed additives

Sung Joon Yu[1], Andrew Morris[2], Advait Kayal[1], Ivan Milošević[3], Thi Thu Hao Van[1,4], Yadav Sharma Bajagai[1] and Dragana Stanley[1§]

[1]Central Queensland University, Institute for Future Farming Systems, Rockhampton, Queensland 4701, Australia

[2]Riverbend Pork Group, 487-489 Ruthven Street, Toowoomba 4350, Australia

[3]University of Belgrade, Faculty of Veterinary Medicine, Bulevar Oslobodjenja 18, 11999 Belgrade, Serbia

[4]School of Science, RMIT University, Bundoora, VIC 3083, Australia

[§]Corresponding author

Dragana Stanley

Central Queensland University

Institute for Future Farming Systems

Central Queensland Innovation and Research Precinct (CQIRP)

Rockhampton QLD 4701, Australia

Telephone: +61 7 49302079

E-mail: D.Stanley@cqu.edu.au

ORCID:

SJY: 0000-0001-8319-50107;

AK: 0000-0002-0048-1516;

YSB: 0000-0002-3043-071X;

DS: 0000-0001-7019-4726

Supplementary Table S1. Performance data of weaner-to-grower pigs fed with and without phytogenic additives.

Age	Measure	Control		Treatment	
W3	Average body weight (kg)	6.486		6.218	
	SD	1.4		1.444	
W8	Average body weight (kg)	20.286		20.382	
	SD	3.647		4.752	
		W3	W8	W3	W8
Maximum body weight (kg)		10.6	29.7	8.9	29.65
Minimum body weight (kg)		3.6	12.9	3.6	11.35
25th percentile		5.4	17.74	5.175	16.98
75th percentile		7.35	22.46	7.1	23.15
Weight gain (kg)		13.80		14.16	

Supplementary Table S2. The SIMPER analysis was conducted on faecal samples at week 8 with contribution of individual genus to the dissimilarity between control and treatment.

Species	Control	Treatment
Prevotella	0.2	0.22
Muribaculaceae	0.08	0.09
Streptococcus	0.04	0.03
Lactobacillus	0.04	0.04
Others	0.1	0.08
Roseburia	0.01	0.03
Clostridium sensu stricto 1	0.03	0.02
UCG-005	0.04	0.03
Faecalibacterium	0.04	0.04
Megasphaera	0.02	0.02
Subdoligranulum	0.05	0.05
Alloprevotella	0.02	0.02
UCG-002	0.02	0.02

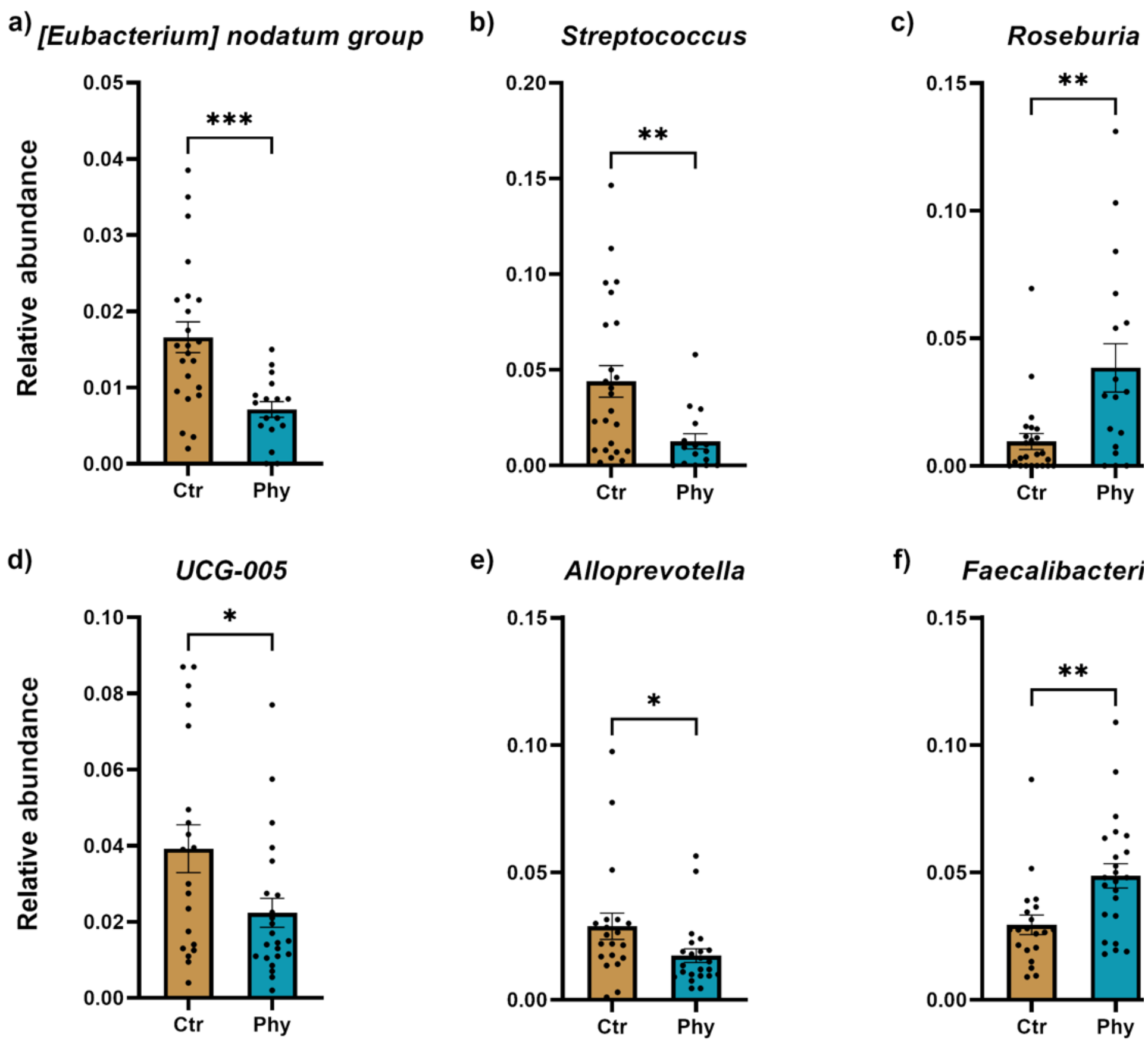

Supplementary Fig. S1 Comparative analysis of genus-level relative abundance in faecal samples from male and female piglets at week 8. Panels a) to c) represents genera differentially abundant in male piglets, while panels d) to f) represent those in female piglets when comparing Ctr to Phy groups. a) *[Eubacterium] nodatum group*, b) *Streptococcus*, and c) *Roseburia* show significant differences in males; d) *UCG-005*, e) *Alloprevotella*, and f) *Faecalibacterium* are represented for females. Significance levels are denoted: * p<0.05, ** p<0.001, *** p<0.0001.

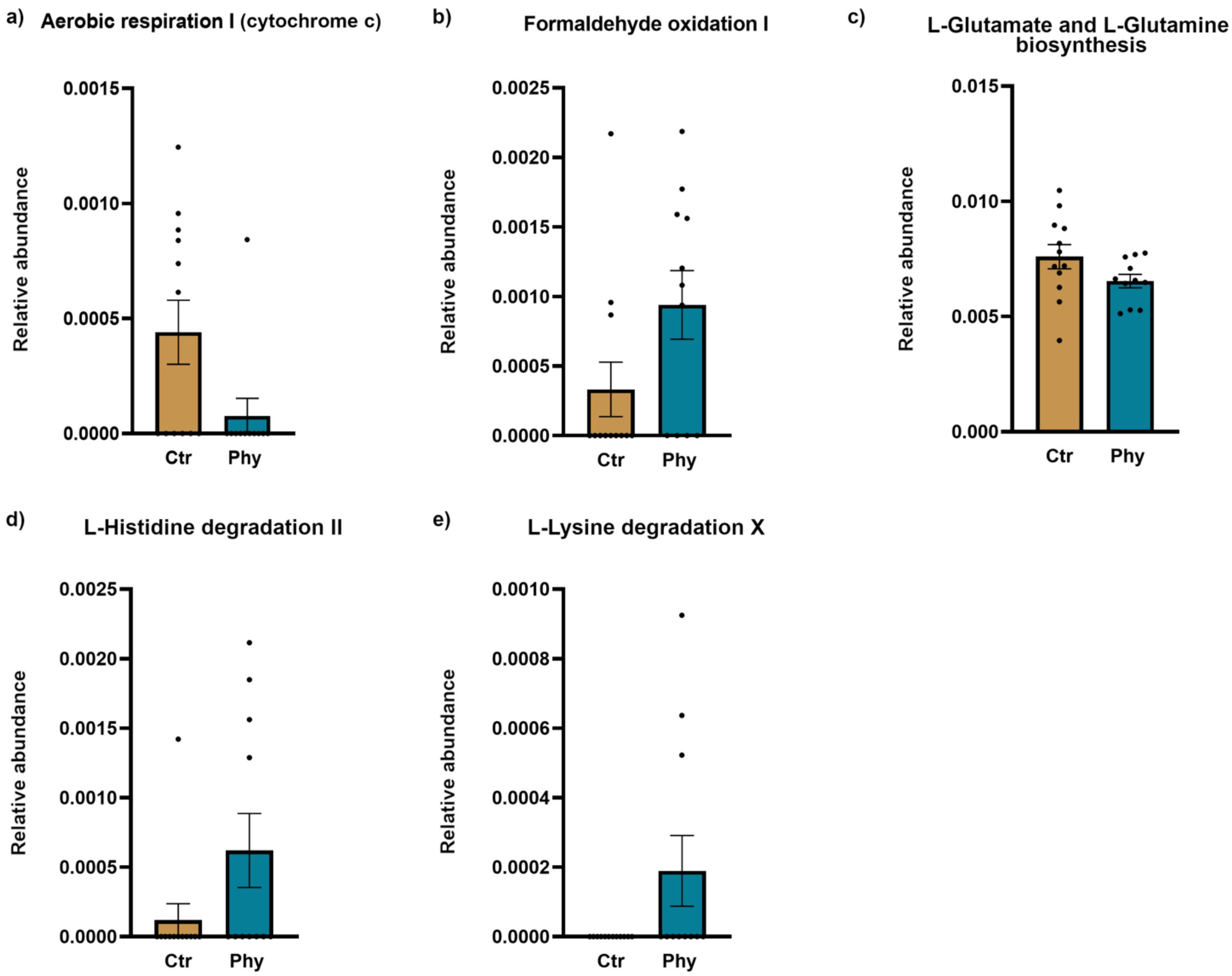

Supplementary Fig. S2 Examination of marginally altered metabolic pathways in metagenomic analysis between Ctr and Phy groups. a) Aerobic respiration I (cytochrome), b) Formaldehyde oxidation I, c) L-Glutamate and L-Glutamine biosynthesis, d) L-Histidine degradation II, and e) L-Lysine degradation X. Each bar represents the mean relative abundance of a pathway, with error bars indicating the standard error of the mean.
